# Enhancing Outcomes and Efficiency in Large Epidermal Cyst Management: Quality Improvement Approach in Primary Care

**DOI:** 10.3390/clinpract14060190

**Published:** 2024-11-12

**Authors:** Waseem Jerjes, Pratik Ramkumar, Yousuf Yaqub

**Affiliations:** 1 Research and Development Unit, Hammersmith and Fulham Primary Care Network, London W6 7HY, UK; 2 Faculty of Medicine, Imperial College London, London SW7 5NH, UK; pratik.ramkumar21@imperial.ac.uk (P.R.); yousuf.yaqub21@imperial.ac.uk (Y.Y.)

**Keywords:** surgical outcomes, clinical efficiency, patient engagement, postoperative care, workflow optimisation

## Abstract

Background: Epidermal cysts are common benign lesions encountered in primary care, especially in minor surgery clinics. The management of large epidermal cysts (>5 cm in diameter) poses significant challenges, including surgical intervention requirements, potential for complications, and impacts on patient care and clinic workflow. The prevalence of these cysts underlines the need for optimised management strategies that are essential for enhancing patient outcomes and clinic efficiency. This quality improvement initiative sought to better manage large epidermal cysts in primary care settings. Patients and methods: The initiative utilised the Plan-Do-Study-Act (PDSA) cycle over three distinct phases, with an emphasis on improving surgical techniques and postoperative care, optimising clinic workflow, and enhancing patient education and involvement. Over the course of this eighteen-month study, 100 patients who required surgical excision of large epidermal cysts were included. The interventions focused on standardising surgical protocols, implementing a new scheduling system, and developing comprehensive educational materials for patients. Results: The programme contributed to major efficiency gains for surgeries: the average operative time was reduced from 45 min to 30. The postoperative complication rate decreased dramatically while patient and clinician satisfaction went up, as did clinic throughput. With patient education enhancements, follow-up adherence rose to 92% while the postoperative complication rate declined from 18% to 9% with the overall approach to patient engagement. Conclusions: The successful application of the PDSA cycles in this work demonstrates that quality improvement methodologies have a potential role in optimising management for large epidermal cysts in primary care settings. Developed interventions can therefore be put into routine care that will indeed improve patient outcome, clinician experience, and operational efficiency in minor surgery clinics.

## 1. Introduction

Epidermoid cysts are benign lesions from the pilosebaceous glands, encapsulated subepidermal nodules filled with keratin. They most often appear on the face, neck, and trunk, but an epidermoid cyst can occur anywhere on the body [1,2,3]. Epidermoid cysts are common dermatological conditions that are easily encountered in primary care settings. Although often thought of as minor, large epidermal cyst (>5 cm in diameter) management is actually a significant challenge encountered in minor surgery clinics, since the condition is quite prevalent and the treatment plan may be associated with complexities [4,5]. Traditional management of large epidermal cysts in primary care often involves unstandardised surgical approaches and variable postoperative care, leading to inconsistent outcomes and higher complication rates. Epidemiologically, while the exact prevalence of large cysts in the UK (>5 cm in diameter) is not thoroughly documented, epidermal cysts overall are quite common. They primarily affect adults between the ages of 20 and 40, with a slight predominance in men. Worldwide, epidermal inclusion cysts account for approximately 85–95% of all excised cysts [6].

Large cysts require surgical intervention, which can have many impacts on patient care and clinic workflow. Prevalence of a cyst, which is usually associated with a host of potential complications, in particular the risks of infection or recurrence, provides the motivation to optimise the management of these disorders by the frontline primary care physicians for better patient outcomes and clinic efficiency.

Large epidermal cysts pose a significant challenge in minor surgery clinics under current practises. Clinicians are faced with challenges in which they have to ensure effective and efficient care due to miscellaneous patient presentation, and variable technical demands in the management of surgical excision. Infections are a frequent presentation, often pre-operatively in these cases. Surgical site infection, especially in regions of high skin tension like the back and neck, remains one of the commonest complications post excisions [2,7]. The variable size and site of the occurrence of cysts, together with patient factors, lead to a wide spectrum of outcomes that very often require tailor-made surgical approaches to address the complications effectively [3,8].

In addition, the procedures will likely impose a heavy workload, bringing about inefficiencies that adversely affect the overall performance of the clinic [9]. Further, suboptimal patient education about the conditions and its management is bound to create potential gaps in patient knowledge and dissatisfaction with care [4,10]. All these factors collectively prevent the delivery of optimum care and drive the need for structured approaches toward better management practises, patient outcomes, and clinician satisfaction [11,12].

The main objective of this quality improvement project is to better manage large epidermal cysts in a primary care environment through a series of specific interventions, following the Plan-Do-Study-Act framework. This framework has shown great efficacy in inducing change across a wide variety of specialties provided there is proper adherence to key methodological factors [11], and is implemented as the basis of our interventions. The interventions themselves focus on enhancing surgical technique, optimising clinic workflow via scheduling, and prioritising patient education and engagement by creating educational materials.

The goals include improving patient outcomes by lowering complication rates and enhancing wound healing, increasing clinician satisfaction by refining surgical techniques and clinic workflows, and improving patient education about the condition and its treatment. By focusing on these critical areas, the project seeks to demonstrate the effectiveness of quality improvement strategies in optimising both patient care and clinic efficiency, particularly within minor surgery clinics.

## 2. Patients and Methods

This quality improvement project was conducted in a primary care setup—North End Medical Centre—from 2020 to 2023, more specifically in a minor surgery clinic to manage a variety of cutaneous and subcutaneous dermatological conditions. There was a total of one hundred patients who participated in this project and underwent excision of large epidermal cysts of the neck and trunk. Patients were referred to our centre from numerous primary care centres within Northwest London. The diagnosis was clinical and patients were referred mainly due to symptomatic causes, including pain, swelling (causing feeling of pressure), and/or recurrent infections. All included patients had the diagnosis of epidermal cyst confirmed histopathologically post excision.

Pre-intervention data were sourced from the routine auditing of our minor surgery procedures prior to the implementation of the PDSA cycle approach and were not part of the 100 patients included in this study. One licenced community-based surgeon (author: W.J.) undertook all procedures and was assisted by a trained nurse (three in total). The single-clinician approach in this initiative was an opportunity for maintaining consistency in technique and care, thus allowing the clear evaluation of outcomes.

Standard care for large epidermal cysts typically involves clinical diagnosis and excision, often without comprehensive pre- or postoperative protocols. In contrast, this study introduced a standardised diagnostic confirmation and a streamlined postoperative protocol, which included pre-emptive use of antibacterial medications and patient education about wound care to reduce complications and improve patient adherence.

The average cyst size in this study was 6.2 ± 0.8 cm, with all cysts being larger than 5 cm in diameter. The incision size varied depending on the cyst size, but the length of the incision was typically slightly longer than the diameter of the cyst, ranging from approximately 6.5 to 7.5 cm, to ensure complete excision while minimising tissue trauma. This approach allowed for full removal of the cyst while reducing the risk of complications, such as incomplete excision or damage to surrounding tissues.

The intervals between the three PDSA cycles were approximately three months each, allowing sufficient time for the implementation and evaluation of each intervention. To optimise workflow, other dermatology cases were scheduled in a staggered manner, prioritising complex cases during quieter clinic times to minimise delays and ensure efficient patient flow.

Antibacterial and analgesic medications were prescribed pre-emptively, a practise that has been adopted as part of the new standardised protocol. The follow-up period was eighteen months, which enabled sufficient time for assessing outcomes throughout the three cycles of the project.

**Figure 1:**
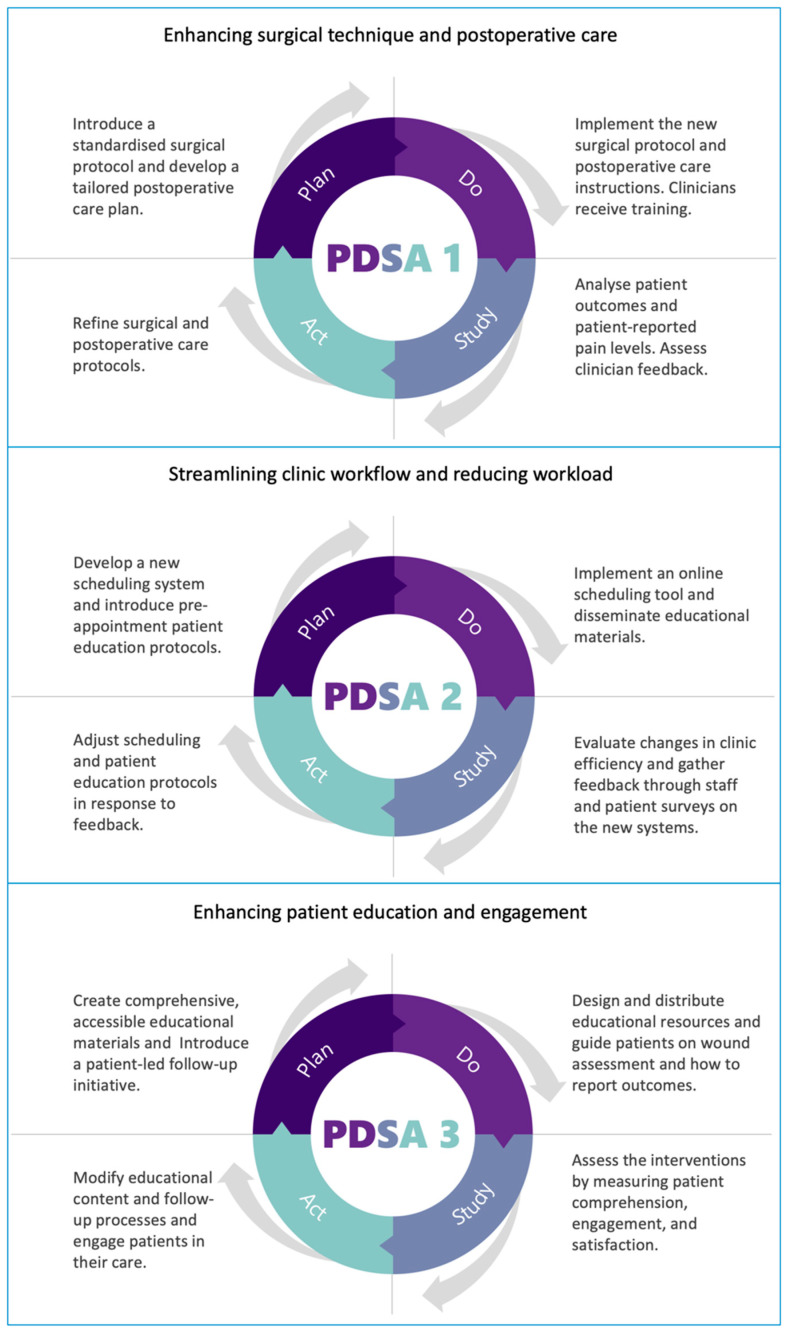
Key processes in quality improvement for large epidermal cyst management: surgical care, workflow optimisation, and patient engagement.

This quality improvement project followed the commonly used iterative model in health settings, the Plan-Do-Study-Act (PDSA) cycle approach (Table 1, Figure 1). This procedure enabled a staged testing of changes in clinical practise: each cycle included planning the change (Plan), doing the change (Do), observing and learning from the consequences of the change (Study), and determining what modifications should be made to the plan (Act). The design of this study adopted the following three PDSA cycles that targeted various aspects of improvement in the management of this cohort of patients presenting with large epidermal cysts: improvement in surgical technique and postoperative care, streamlining of clinic workflow, reduction in workload, and improvement in patient education and engagement. The iterative nature of this design has helped in the development of interventions based on real-world feedback and outcomes in such a way that each cycle built upon the insights gained from its predecessors.

### 2.1. PDSA Cycle 1: Enhancing Surgical Technique and Postoperative Care

The initial phase of our quality improvement project focused on refining the surgical technique and postoperative care to enhance patient outcomes and clinician experience. This included the implementation of a standardised surgical protocol (Appendix A) to reduce operative times and enhance wound-healing outcomes. A concomitant tailored plan for postoperative care was prepared, which accentuated careful wound care and promoted early mobilisation to prevent additional complications.

Implementation of these protocols commenced promptly, along with detailed instructions on the surgical procedures, incisions that would be used, how to remove the cyst, and how to suture in place. The postoperative instructions specified the dressing changes, activity levels, and signs of infection to watch for. Adequate education was delivered to the nursing team implementing the new protocols to ensure the standardisation and fidelity of the protocols for every case.

To evaluate how effective these interventions were, we analysed various patient outcomes, such as how quickly wounds healed, the frequency of complications after surgery, and the levels of pain reported by patients. We also gathered feedback from the clinical team to determine how easy it was to implement the new protocols and how they influenced the efficiency of surgeries. Additionally, we reviewed operational data, including any changes in the time it took to perform surgeries and the overall flow of patients through the clinic, to assess the wider effects of these interventions.

Based on the findings, both the surgical and postoperative care protocols were revised. These revisions were specifically targeted to address any gaps or challenges identified, with the goal of making the interventions as effective and user-friendly as possible.

### 2.2. PDSA Cycle 2: Streamlining Clinic Workflow and Reducing Workload

The second cycle aimed at optimising clinic workflow and reducing the workload on staff, thereby improving overall efficiency and satisfaction. The strategy was to put in place a new scheduling process with the aim of enabling patient flow in the clinic (Appendix B). In addition, a pre-appointment patient education protocol was implemented to digitally share procedural information, expected results, and preparation content.

These strategies were executed with a scheduling tool to have patients choose the appointments that matched their availability while allowing the clinic staff to effectively manage their schedules. Secondly, patient education materials were prepared and shared with patients to prepare them with knowledge before their appointments.

These interventions were assessed for their successes on changes in clinic efficiency, measured by the reduction in waiting time of the patients and an increase in the throughput of the patient. Staff satisfaction surveys were conducted to address reactions toward the new scheduling system, and for the patients to establish readiness to undergo surgery and satisfaction with pre-appointment education.

Adjustments to the scheduling and patient education protocols were made in response to the feedback received, with the goal of further enhancing clinic workflow and patient experience.

### 2.3. PDSA Cycle 3: Enhancing Patient Education and Engagement

The final cycle was committed to better patient understanding and involvement, where there could be more comprehensive understanding of the condition and its management, with the patients being actively involved in postoperative care. This translated to the development of understandable and accessible full journey educational materials from diagnosis to recovery. In addition, the patients were encouraged to take part in postoperative care through a patient-led follow-up initiative.

Comprehension friendliness was considered when educational materials were developed, in printed brochures and an online form. They disseminated information regarding the nature of epidermal cysts, surgical procedure, postoperative care instructions, and warning signs of complications. Patients were also empowered to spearhead the follow-up through wound assessment where they were advised on when to seek medical assessment and how to report back on the outcomes.

Patient understanding, engagement, and satisfaction with these interventions were tested through surveys and interviews. Also, the effect on the rates of adherence to follow-ups and complications was measured to determine any benefit from the burden of increasing patient involvement in their care. Based on the outcomes, alterations were carried out in the curriculum content and follow-up processes so that they met patient needs and helped in effective engagement.

### 2.4. Ethical Considerations

The quality improvement initiative aimed at optimising the management of large epidermal cysts in primary care did not require an ethical review. Quality improvement projects, such as ours, which seek to refine existing clinical practises through Plan-Do-Study-Act (PDSA) cycles, are generally classified as service evaluations. These projects are intended to implement evidence-based practises in line with current ethical standards of care and do not introduce new, experimental treatments or collect patient data beyond what is typically required in clinical settings.

Furthermore, the interventions implemented were part of an ongoing effort to improve both patient care and the operational efficiency of the clinic. These interventions were based on established clinical guidelines and best practises, ensuring that the highest priorities were patient safety and quality of care. Changes were made in areas such as surgical techniques, postoperative care, clinic workflow, and patient education, all with the aim of elevating the standard of care. Patient consent for treatment was obtained as per the usual protocols within routine clinical practise.

Clinical images were not included in this quality improvement project as obtaining consent for images was not part of the original study design. Future studies may include clinical imagery, pending appropriate patient consent, to visually demonstrate surgical outcomes.

## 3. Results

The cohort was evenly divided between males and females and ranged from 18 to 65 years of age, with a mean of 37.5 years. This would provide a good mix; it is an advantage to base an assessment on the intervention with patients across a wide spectrum of demography.

In the first PDSA cycle (Table 2), the new standardised surgical protocol produced a marked decrease in operative times from an average of 45 min per surgery to 30 min. This was the result of simple incision patterns and simplified strategies in cyst excision that curtailed tissue trauma, thus being favourable for wound healing. Postoperative care was significantly improved through the use of individualised care plans, mainly emphasising close wound care and early mobilisation. Complications in the postoperative period therefore were drastically lowered in percentage points: infection rates fell from 10% to 2%, and rates of wound dehiscence fell from 5% to 1%. In addition, patient-reported pain levels using the pain score on a scale of 0 to 10 (where 10 means the most pain) showed significant improvement, with average scores decreasing from 5 to 2 in the postoperative period.

Patients expressed very high levels of satisfaction with the care they received, emphasising the clarity of the postoperative instructions and the shortened recovery times. Specifically, 98% of patients reported that the educational materials provided were useful, and 95% felt confident in managing their care at home. Equally positive was the feedback from the clinical team who felt much more satisfied with the surgical process.

In the second PDSA cycle (Table 3), the new appointment system helped create a better flow of patients, thus reducing the waiting time from 35 min on average to 15 min. This was an improvement measured by six months’ clinic operational data before the intervention and that after the implementation of the intervention.

The number of patients seen per clinic increased by 20%, from an average of 5 to 6 patients, due to the new scheduling system and pre-appointment patient education, which had cut unnecessary time on routine inquiries and clarifications.

The distribution of workload reported by the clinical team (including one doctor and three nurses) became more evenly distributed, with a 30% decrease in the number of reports indicating that they felt overwhelmed, according to a survey conducted before and after the intervention.

The introduction of digital platforms for patient education saw an 80% compliance rate with pre-appointment instructions, up from 50% when information was provided solely during clinic visits. Post-intervention surveys indicated a 25% increase in patient satisfaction regarding their preparedness for the procedure, with 95% of patients rating their satisfaction as “high” or “very high”.

According to the clinical team, there was a significant reduction in the stress they attributed to care coordination and scheduling of their patients. More time was spent in caring for patients as opposed to juggling care coordination and scheduling. This allowed for solid quality care, where more attention was spent on quality over quantity. The online scheduling system, along with the pre-appointment educational information patients received, made it easier to schedule an appointment and the clinics more available with more open appointments, according to one hundred patients. This was seen as allowing the patient to “see what I see” and increases their apparent control over their care experience.

In the third PDSA cycle (Table 4), there was a substantial increase in the average patient understanding score, which evaluated patients’ comprehension of their medical condition, the surgical procedure, and postoperative care. The score almost doubled, rising from 4.5 to 8.7 on a scale of 1 to 10. This significant improvement was paralleled by an increase in patient satisfaction scores, which grew from an average of 6.2 to 9.1. These results highlight the effectiveness of the implemented educational materials and strategies, leading to a more informed and reassured patient population.

The recommended follow-up improved from 65% to 92%; this is a marked rate of adherence and definitely counts as successful patient engagement that the patient-led follow-up initiative has managed to achieve. The overall postoperative complication rate fell from 18% to 9%. This denotes better postoperative care and monitoring for better-educated and more engaged patients.

The implementation of detailed educational resources and the active participation of patients in their postoperative care has greatly improved patient engagement, as evidenced by the average score increasing from 5.8 to 9.4. Here, patients felt more involved in their care process, which likely contributed to the observed rise in follow-up adherence and the reduction in complication rates.

## 4. Discussion

### 4.1. Analysis of Outcomes

The reflective outcomes in this quality improvement initiative ensure that the PDSA framework is valued during the process of improving the management of large epidermal cysts within a primary care setting. Measurable improvements from each cycle targeted specific challenges identified at the outset, with measured improvements reflecting the efficacy of the interventions applied.

The area for improvement in PDSA cycle 1 was surgical skills and postoperative care. The standardised protocol led to improvements in both efficiency and clinical outcomes, reinforcing the benefits of structured approaches to surgical care, thus emphasising that technical efficiency and consistency in practise are very important. A structured postoperative care plan contributed to significant reductions in postoperative complications, highlighting the importance of early mobilisation and vigilant wound management [8]. This cycle directly addressed the importance of the objective to improve patient outcomes and the critical place that surgical and postoperative protocols have within patient care.

The second PDSA cycle focused on improving clinic flow and decreasing the workload of the clinicians and staff. The clinic was functioning more efficiently due to the impact of the changes proposed, with operational efficiencies improved notably due to the new appointment system, reflected in shorter waiting times and enhanced patient flow. This reduced operational inefficiency, increased clinician satisfaction and gave way for a more patient-centred model of care. This cycle demonstrated that improvements in operations can create a massive impact on both the performance of the clinic and the experience of the patients themselves.

PDSA cycle 3 focused on enhancing patient education and engagement. The cycle entailed broad, comprehensible access to all patient educational materials, as well as a follow-up programme developed by the patient. This significantly improved patient understanding, satisfaction, and involvement. Patient education and involvement enhance the patient’s experience of care and its outcome [9,13].

Traditional management of epidermal cysts often lacks a cohesive, standardised approach, particularly in primary care settings. This inconsistency can lead to variability in patient outcomes, as surgical techniques and postoperative care are not uniformly applied. The absence of structured follow-up protocols further exacerbates this issue, as patients may not receive consistent guidance on postoperative care, resulting in suboptimal recovery experiences and lower levels of satisfaction.

By contrast, the updated management process introduced in this study is rooted in a more structured and patient-centred approach. The standardisation of surgical protocols ensures that all patients receive consistent, high-quality care, reducing the potential for variability in outcomes. This consistency is particularly important in primary care settings, where clinicians may be managing a broad range of conditions. The integration of proactive postoperative care, which emphasises patient education and engagement, not only improves the clinical outcomes but also empowers patients to take a more active role in their recovery. This empowerment fosters a sense of ownership over their health, which can significantly enhance patient satisfaction and adherence to care plans.

Operationally, the updated approach helps streamline clinic workflows by addressing inefficiencies in scheduling and patient communication. By incorporating digital tools and pre-appointment education, the new process reduces the burden on staff while ensuring that patients are well-prepared for their procedures. This shift towards a more technology-enabled and patient-informed model aligns with broader trends in healthcare towards efficiency and sustainability. Additionally, the holistic focus on both clinical and operational improvements makes the updated process more adaptable to the complexities of modern healthcare environments, where balancing patient care with workflow efficiency is critical.

### 4.2. Implications for Practise

The results of this quality improvement project have numerous implications for routine care in primary care settings: first, standardising surgical and postoperative care protocols can go a long way in increasing patient outcomes and clinician satisfaction. Such protocols ensure the consistent delivery of care, reduce the risk of complications, and improve efficiency in clinical operations. Furthermore, as shown by Pucher et al. [10], protocol-led care is an example of clear, evidenced-based delivery of care which is deliverable at a local level without structural or organisational changes, having the potential to be more cost-effective.

Another operational challenge that will need to be addressed is patient experience and staff satisfaction. This, again, can be enhanced through the use of digital tools in scheduling and education. Such tools offer an easily scalable system of managing patient flow and pre-appointment preparation—both of which are critical in high-volume primary care settings. A Health Consumer Powerhouse survey showed that patients also experienced better ease of appointment in countries where “in other words, resources are seen as being reasonably stretched” like Austria. They have also helped to increase satisfaction among staff since they reduce the level of administrative work that they have to perform and make patient interaction easier. Empirical evidence clearly indicates that by implementing these technologies, one does not only improve the efficiency of the workflow but also enhances clinical outcomes by promoting better engagement with patients, adherence to treatment protocols, and overall satisfaction [11,12].

Finally, empowering patients through comprehensive education and active engagement not only enhances their understanding and satisfaction but also leads to better outcomes after surgery. Involving patients in the care process is essential in a patient-centred care model, as it contributes to better health outcomes and improves the quality of services provided [13]. Additionally, educating patients helps reduce their anxiety and increases their confidence in the care they receive, which supports their psychological and emotional well-being both before and after surgery. This is crucial for achieving positive postoperative outcomes, as lower stress levels have been linked to faster healing and fewer complications, such as infections or delayed wound healing [13].

### 4.3. Limitations

The principal limitations of this study relate to the sample size and the setting. Generalisation is limited by conducting the initiative on one hundred patients and within only one primary care setting. The special features of the patient population, resources, and dynamics of the clinic may not be generalisable to other primary care settings, making the interventions less applicable in other contexts.

In addition, the study was conducted by one operating clinician, which, although ensuring consistency in surgical techniques and care, limits the generalisability of the findings. While this approach allowed for a controlled environment and consistent application of the protocols, it introduces variability in how the results might translate across a broader range of clinicians with differing levels of experience and technical skill. Surgical outcomes, patient engagement, and adherence to postoperative care protocols could vary significantly depending on the individual clinician’s skill set and familiarity with standardised approaches. Furthermore, the single-clinic setting further restricts the ability to apply these findings across different healthcare systems and clinic environments.

To enhance the robustness and applicability of the results, future studies should involve multiple clinicians across various clinical settings. Such multi-clinician studies would provide valuable insights into how well the standardised protocols perform when implemented by different providers with varying levels of expertise. By involving a diverse group of practitioners and clinic environments, future research would yield more reliable and universally applicable findings, offering greater confidence in the scalability of the interventions across the broader healthcare spectrum.

### 4.4. Future Directions

Future research should replicate this study across various primary care settings with a larger and more diverse patient cohort to assess the scalability and generalisability of the interventions. Moreover, investigating the integration of advanced technologies, such as telemedicine for postoperative follow-ups, could provide further insights into improving patient care and clinic efficiency. Additional PDSA cycles might focus on addressing specific challenges identified during this initiative, such as improving clinician training on standardised protocols and creating more targeted patient education materials for diverse populations.

Moreover, implementing similar initiatives across various primary care clinics, each with diverse patient populations, resources, and operational structures, would yield more generalisable data. This broader approach would facilitate a better evaluation of the intervention’s scalability and effectiveness in different settings. Furthermore, adopting a multi-clinician strategy is crucial for understanding how differences in clinician skill, experience, and adherence to standardised protocols impact the outcomes. This approach would provide valuable insights into whether the benefits observed in this study are consistent across a wider range of clinicians, ensuring that the interventions are robust and applicable across diverse healthcare environments.

Therefore, the potential for this quality improvement initiative to demonstrate how targeted interventions, within the framework of PDSA, could substantially improve the management of large epidermal cysts in primary care is clearly shown. The iterative nature of the interventions with a focus on surgical efficiency, clinic operations, and patient education makes this approach comprehensive in improving outcomes, clinician satisfaction, and operational efficiency.

## Figures and Tables

**Table 1:** Optimising surgical and clinical practises through standardised protocols, workflow enhancements, and patient engagement strategies.

PDSA Cycle	Plan	Do	Study	Act
Enhancing surgical technique and postoperative care	Introduce a standardised surgical protocol aiming to reduce operative times and improve wound healing. Develop a tailored postoperative care plan focusing on meticulous wound care and encouraging early mobilisation.	Implement the new surgical protocol and postoperative care instructions. Clinicians receive training to ensure consistency across all cases.	Analyse patient outcomes including wound healing times, incidence of postoperative complications, and patient-reported pain levels. Assess clinician feedback on the adoption of the new protocols and its impact on surgical efficiency.	Refine surgical and postoperative care protocols based on findings, addressing identified gaps and challenges to improve effectiveness and user-friendliness.
Streamlining clinic workflow and reducing workload	Develop a new scheduling system to enhance patient flow and reduce staff workload. Introduce pre-appointment patient education protocols using digital platforms.	Implement an online scheduling tool and disseminate educational materials to patients via email and the clinic’s website.	Evaluate changes in clinic efficiency (e.g., reduced waiting times, increased throughput) and gather feedback through staff and patient surveys on the new systems.	Adjust scheduling and patient education protocols in response to feedback to further enhance clinic workflow and patient experience.
Enhancing patient education and engagement	Create comprehensive, accessible educational materials covering the care journey. Introduce a patient-led follow-up initiative for active postoperative care participation.	Design and distribute educational resources in various formats. Guide patients on wound assessment and how to report outcomes through the follow-up initiative.	Assess the interventions by measuring patient comprehension, engagement, and satisfaction, along with the impact on follow-up adherence and complication rates.	Modify educational content and follow-up processes based on outcomes to ensure they meet patient needs and effectively engage them in their care.

**Table 2:** Impact of intervention on surgical outcomes: pre- and postoperative analysis.

Outcome Measure	Pre-Intervention	Post-Intervention	Percentage Improvement
Average operative time (minutes)	45	30	33.33%
Incidence of postoperative infections (%)	10	2	80%
Incidence of wound dehiscence (%)	5	1	80%
Average patient-reported pain level	5	2	60%

**Table 3:** Effectiveness of workflow enhancements: pre- and postoperative outcomes in clinic efficiency and patient satisfaction.

Outcome Measure	Pre-Intervention	Post-Intervention	Change (%)
Average waiting time (minutes)	35	15	−57.14
Average number of patients per clinic	5	6	+20
Clinician workload complaints	60%	30%	−50
Compliance with pre-appointment instructions	50%	80%	+60
Patient satisfaction (preparedness)	70%	95%	+35.71

**Table 4:** Impact of patient education and engagement interventions: pre- and postoperative metrics analysis.

Metric	Pre-Intervention	Post-Intervention	Change (%)
Patient understanding score (1–10)	4.5	8.7	+93.3%
Patient satisfaction score (1–10)	6.2	9.1	+46.8%
Follow-up adherence rate	65%	92%	+41.5%
Postoperative complication rate	18%	9%	−50%
Patient engagement score (1–10)	5.8	9.4	+62.1%

## Data Availability

The datasets generated and/or analysed during the current quality improvement project are not publicly available due ethical reasons but are available from the corresponding author upon reasonable request.

## Appendix A

Standardised surgical protocol.

- Pre-operative preparation:
  - Comprehensive patient evaluation to assess suitability for in-clinic excision.
  - Patient education on procedure, expected outcomes, and postoperative care.
  - Informed consent detailing potential risks and benefits.
- Surgical technique:
  - Use of aseptic technique throughout the procedure.
  - Local anaesthesia administration with lidocaine (with or without epinephrine) to ensure patient comfort.
  - Minimal incision approach to excise cyst in toto, aiming to reduce tissue trauma and scarring.
  - Meticulous haemostasis using monopolar diathermy as necessary.
  - Closure technique selection based on incision size and location, utilising either absorbable sutures for deeper tissue layers or non-absorbable sutures for skin closure.
- Postoperative care:
  - Detailed postoperative instructions covering wound care, activity restrictions, and signs of infection or complications.
  - Prescription of analgesics for pain management, if necessary.
  - Schedule follow-up appointment for wound assessment and suture removal.
- Documentation and data collection:
  - Accurate and comprehensive recording of procedure, findings, and any complications in patient’s medical record.
  - Collection of data on operative times, complication rates, and patient satisfaction for ongoing quality improvement analysis.

## Appendix B

The new scheduling system introduced as part of a quality improvement project for wound care management represents a significant step forward in enhancing patient access and clinic efficiency. During the development phase, a comprehensive analysis was conducted to identify the inefficiencies plaguing the existing scheduling system, particularly as they impacted wound care management. This phase engaged a wide range of stakeholders, including clinicians, administrative staff, and patients, to gather insights and pinpoint specific challenges. The incorporation of technology stood out as a pivotal component, facilitating online appointment booking, automated reminders, and real-time updates on clinic wait times. This approach aimed not only to improve accessibility for patients but also to alleviate the administrative burden on clinic staff, thus optimising patient flow and the overall clinic environment for wound care.

The implementation phase of the new scheduling system was characterised by a phased rollout, which allowed for iterative feedback and necessary adjustments based on real-world usage. Initially focusing on a pilot group within the clinic, this strategy facilitated fine-tuning the system, ensuring its effectiveness in a practical setting. Continuous monitoring and evaluation during this phase was key to assessing the impact of the scheduling system on clinic workflow and patient satisfaction, thereby guiding ongoing improvements and adjustments to optimise the system’s effectiveness.
